# The protocol of DECO-MOM: a clinical trial testing the effectiveness of a mobile application for an environmental health intervention among pregnant women

**DOI:** 10.1186/s12911-023-02258-5

**Published:** 2023-08-09

**Authors:** Hyun Kyoung Kim

**Affiliations:** Department of Nursing, Kongju National University, Gongju, South Korea

**Keywords:** Environmental health, Women, Pregnancy, Health behaviors, Mobile application

## Abstract

**Background:**

Environmental toxins are particularly harmful to pregnant women and their fetuses due to the long-term effects of these toxins on children after birth. Environmental health behaviors can prevent and protect mothers and their babies’ environmental health.

**Methods/Design:**

This study presents a protocol for a double-blinded randomized controlled trial to examine the effect of a mobile application named “DECO-MOM” (Deep ECOlogy-MOM) for pregnant women. This application contains content related to environmental perceptions and behaviors according to the revised protection motivation theory. The mobile intervention will be implemented for 4 weeks for 40 pregnant women with a gestational age in the first trimester. As the control group, 40 pregnant women will be recruited at public healthcare centers in Chuncheon and Gongju in South Korea. The hypothesis is that the experimental group will have higher scores for environmental perceptions and behaviors than the control group. An online survey administered before and after the intervention will contain outcome parameters including depression, stress, quality of life, e-learning satisfaction, environmental perceptions, and environmental behaviors.

**Discussion:**

This study will elaborate a guide for an intervention to enhance the effects of the mobile application on strengthening environmental health perceptions, behaviors, e-learning satisfaction, and quality of life and curtailing depression and stress among pregnant women.

**Trial registration:**

Trial registration Number: KCT0007725, Registered September 22, 2022. Prospectively registered.

## Background

Environmental health is defined as the protection of the human body from environmental toxins such as air, climate, drinking water, chemicals, and radiation to maintain safety and wellness [1]. Environmental health hazards include exposure to substances, stimuli, or environmental conditions such as toxic substances and stressors. The characteristics of an exposure include the concentration, time, duration, frequency, and route of contact [2]. Pregnant women are a vulnerable population to environmental exposures because estrogen receptors are more widely distributed in the reproductive organs, breasts, and adipose tissue than before pregnancy. The placental tissues transfer environmental toxins to the fetus and affect the formation and growth of human organ systems, especially in the first trimester [3]. Climate change and air pollution exacerbate and induce health problems in pregnant women from micro-dust, ozone, and heavy metals [4].

Environmental toxicants such as endocrine disruptors (EDCs) affect pregnant women, fetuses, neonates, infants, and children throughout the lifespan. Correlations have been reported between lead in the water and a low birth rate [5] and between other chemicals with a low fertility rate [6]. Air pollution has been reported to show correlations with preterm birth and spontaneous abortion [7]. In fetuses, associations have been identified between phthalates and small for gestational age [8], between lead exposure and fetal death [5], and between EDCs and congenital anomalies [6]. In newborn babies, environmental pollutants have been reported to be correlated with low birth weight [5, 8–10]. In infants, impacts of environmental pollutants on atopic dermatitis, respiratory diseases, cognitive delays, attention deficit disorder, and sleep disturbances have been reported [10], as well as a correlation between phthalates and male reproductive disorders [11]. In children, correlations have been reported between phthalates and mental development delays [12], low weight, and low body mass index [8], as well as between electric wave exposure and cognitive delay [13].

Preventing environmental exposure and protecting the health of pregnant women and their babies are important for the health status of the next generation given the circumstances of climate change and environmental pollution [14]. Birth education about environmental health is the principal vehicle to convey information to pregnant women and their family members about health behaviors, and internet-based interventions are useful in the post-pandemic society [15]. Among various approaches to internet-based interventions, mobile applications are particularly feasible and accessible [16]. However, few studies have explored the possibility of promoting environmental health behaviors among pregnant women via a mobile application intervention, although one study was conducted on an intervention using online lectures and cartoon comics [17]. Hence, this study will be aimed to test the effectiveness of a mobile application intervention named “DECO-MOM” (Deep ECOlogy-MOM) for pregnant women. The hypothesis of this study is that this mobile application intervention for pregnant women will enhance their environmental perceptions and behaviors. Additionally, this study will test e-learning satisfaction, depression, stress, and quality of life as secondary outcomes.

## Methods

### Study design

This study will have a 1:1 parallel randomized controlled trial design. The researchers and participants will be double-blinded during the registration, allocation, intervention, and statistical analysis phases. Cluster sampling will be used with blocked randomization at two health centers. The intervention group and control group will be recruited from registration lists for birth education by two trained research assistants at health care centers using SPSS for Windows version 24.0 (IBM Corp., Armonk, NY, USA) to conduct randomization. Participants’ identities will be coded to ensure that they remain unknown to the researchers. The participants will be pregnant women in the first trimester who have no physical or psychological diseases. This study will receive approval from the institutional review board of Kongju National University, and the participants will complete written consent forms in face-to-face meetings with the researchers. This protocol was developed and documented in accordance with the Consolidated Standards of Reporting Trials (CONSORT) guidelines [18] and registered in the Korean Clinical Research Information Service [19]. The registration number (KCT0007725) can be retrieved from https://cris.nih.go.kr/cris/search/detailSearch.do?seq=22378&status=5&seq_group=22378&search_page=M (22/09/2022).

### Setting

We will contact the public health care centers of Chuncheon, a city in Gangwon Province, and Gongju, a city in Chungcheongnam Province, in South Korea. These centers operate maternal child clinics that provide pregnancy nursing services for pregnant women. These two cities are similar in terms of prenatal education, rural-urban characteristics, total fertility rate (Chuncheon: 0.96; Gongju: 0.97) [20], and average microdust (particulate matter 10) density (Chuncheon: 21.5 mg/m^3^; Gongju: 26.3 mg/m^3^) [21], helping to alleviate concerns about potential bias. Maternal child clinic nurses will provide brochures to women in the first trimester to invite them to register for prenatal education.

### Inclusion and exclusion criteria

The inclusion criteria will be (1) pregnant women in the first trimester who are (2) aged over 18 years, (3) can write and speak in Korean, (4) have one or more smart gadgets (smartphone, notebook, or tablet), and (5) want to receive birth education for 4 weeks. The exclusion criteria are (1) hospitalization due to a disease and (2) currently receiving treatment or medication for a disease.

### Allocation

In total, 40 participants (20 recruited from each of the two cities) will be allocated to the intervention group (mobile application plus usual prenatal class) and 40 participants (20 from each of the two cities) will be allocated to the control group (usual prenatal class only). If pregnant women agree to participate in the study, nurses at the centers will obtain their mobile phone numbers and refer them to the researcher. The researcher will contact the pregnant women and explain the study’s purpose, content, intervention, benefits, and ethical considerations. The final participants will receive the intervention for 4 weeks via a mobile application and complete pre-post surveys.

### Intervention

#### Intervention group

DECO-MOM will be created for the Android operating system, with a user interface (UI) and user experience (UX) designed in a user-friendly and interactive manner by professionals in the field of application development. The researchers will communicate with the developers about the goals, titles, contents, strategies, animation, graphics, icons, banners, other aspects of technology, launch, updates, and sustainable testing. The web application interface will contain daily notifications, weekly feedback, and linked videos made by the researchers. The researchers will create a prototype using the application maker program “KakaoOven” and will conduct quality assurance testing through the Mobile Application Rating Scale (MARS) [22], a standardized scale for mobile application validation. The MARS contains items on basic characteristics, quality appraisal (engagement, functionality, aesthetics, and information), subjective appraisal, and purpose of using the application. It has a total of 23 items scored on a 5-point Likert scale from 1 point (inappropriate) to 5 points (very excellent). The internal consistency reliability of the MARS was demonstrated by a Cronbach’s alpha value of 0.90.

The 4-week DECO-MOM intervention will be conducted via a mobile application. A research assistant will contact participants by phone before the intervention and explain the program. The intervention will consist of (1) helping participants understand the severity of environmental toxins and susceptibility during pregnancy, (2) motivating participants to engage in environmental health behaviors and enhancing their self-efficacy, (3) enhancing perceptions of the benefits of health behaviors and working to overcome the perceived costs, and (4) empowering personal environmental behaviors and communal behaviors each week. Participants will be able to access DECO-MOM through a link or QR code provided through the KakaoTalk social networking service, which is ubiquitously used in Korea. A trained research assistant will personally contact each participant by phone every week for encouragement, and if there are open-ended questions about the application, a research assistant will provide coaching via a phone call lasting at least 10 min for each participant in DECO-MOM (Table 1).

**Table 1:** Contents of the DECO-MOM intervention program

Sessions	Themes	Intervention format	Additional treatment
First week	Understanding of the severity of environmental toxins and susceptibility during pregnancy	Mobile application Gamification	Telephone coach
Second week	Motivation of environmental health behaviors and enhancing self-efficacy	Mobile application Encouragement	Telephone coach
Third week	Perception of benefits of health behaviors and overcoming cost	Mobile application Empowerment	Telephone coach
Fourth week	Empowering personal environmental behaviors and communal behaviors	Mobile application Embodiment	Telephone coach

Topics of the mobile application for environmental health intervention

The DECO-MOM program will be developed based on the revised protection motivation theory (rPMT). According to the rPMT [23], when humans feel fear in a dangerous situation, they make an internal evaluation. There are four categories of internal mechanisms of perception: perceived danger to one’s health (severity), one’s perception that a situation can cause health problems (susceptibility), the belief that one’s actions will help to protect one’s health (response efficacy), and the perceived capability that one can change one’s health behaviors (self-efficacy). Cognitive processes influence health behaviors through the formation of behavioral intentions, and when one feels that the benefits of a behavior outweigh the disadvantages, a behavior change can occur [23].

#### Control group

For the control group, treatment as usual will be provided via face-to-face education for 4 weeks. Each session will be conducted in the birth class room in the public health center for 2 h by a midwife. The content will be about (1) understanding pregnancy and fetal growth; (2) birth pain control, relaxation, and breathing during labor; (3) postpartum care and breastfeeding; and (4) newborn baby care. Participants will be recruited in four rounds to ensure sufficient numbers.

### Response to participants

Withdrawal from the mobile application-based intervention can occur because of personal schedule-related reasons, disease, or moving to another region. To avoid withdrawal from the intervention, personal contact will be continued via social networking services every week. In case of withdrawal, the reasons will be assessed to examine the drop-out effect.

### Assessments

The pre- and post-test will be assessed just before and after the intervention. For both groups, the pre- and post-test will be conducted via online survey using a Naver Form distributed personally through the KakaoTalk messenger. The online survey will take 10 min. After the pre- and post-test, an online gift worth 5 dollars will be provided to the participants. The survey will take 10 min, and a gift worth 5 dollars will be provided to the participants after the pre- and post-test. The questionnaire will contain items on participants’ general characteristics (age, gestational age, gravidity, parity, the presence of a living baby, past medical history), environmental perceptions, environmental behavior, depression, anxiety, quality of life, and e-learning satisfaction. The general characteristics will be surveyed only in the pre-test, and e-learning satisfaction will be surveyed only in the post-test (Table 2).

**Table 2:** Study assessments and time points

	WEHP	WEHB	EPDS	GAD-7	EQ-5D	e-LSS
Pre-test	x	x	x	x	x	
Post-test	x	x	x	x	x	x

Primary outcomes: WEHB (Women’s Environmental Health Behavior) and WEHP (Women’s Environmental Health Perception)

Secondary outcomes: GAD-7 (General Anxiety Disorder-7), e-LSS (e-Learning Satisfaction Scale), EPDS (Edinburgh Postpartum Depression Scale), and EQ-5D (European Quality of life 5-Dimension)

### Outcome measurements

#### Primary outcomes

##### Environmental health perceptions

Environmental health perceptions will be measured using the Women’s Environmental Health Perception (WEHP) scale [24]. The WEHP consists of the following domains: severity (10 items), susceptibility (11 items), response efficacy (10 items), self-efficacy (14 items), benefit (8 items), and barriers (10 items). Responses to the WEHP scales are measured using a Likert scale, ranging from 1 (not at all) to 5 (strongly agree). The Cronbach’s alpha values for internal consistency reliability were 0.84 for severity, 0.92 for vulnerability, 0.88 for response efficacy, 0.90 for self-efficacy, 0.91 for benefits, and 0.85 for barriers.

##### Environmental health behaviors

Environmental health perceptions will be measured using the Women’s Environmental Health Behavior (WEHB) scales [24]. The WEHB consists of personal health behavior (14 items) and community health behavior (16 items). Responses to the WEHB scales are measured by a Likert scale, ranging from 1 (not at all) to 5 (strongly agree). The Cronbach’s alpha values for internal consistency reliability were 0.90 for personal health behavior and 0.91 for community health behavior.

#### Secondary outcomes

##### Depression

Depression will be measured using the Edinburgh Postpartum Depression Scale (EPDS) [25]. The Korean version of the EPDS (10 items) will be used [26]. Responses to the EPDS are measured using a Likert scale, ranging from 1 (frequently) to 4 (not at all). Cronbach’s alpha for internal consistency reliability was 0.87.

##### Anxiety

Anxiety will be measured using the General Anxiety Disorder-7 (GAD-7) [27]. The Korean version of the GAD-7 (7 items) will be used [28]. Responses to the GAD-7 are measured using a Likert scale, ranging from 1 (not at all) to 4 (nearly every day). Cronbach’s alpha for internal consistency reliability was 0.97.

### Quality of life

Quality of life will be measured using the EuroQol-5D (EQ-5D) [29]. The Korean version of the EQ-5D (5 items) will be used [30]. Responses to the EQ-5D scale are measured using a Likert scale, ranging from 1 (not at all) to 4 (nearly every day). The intraclass correlation (kappa) for inter-rater reliability was 0.61 (95% confidence interval 0.46-0.72) [30].

### E-learning satisfaction

E-learning satisfaction will be measured using the E-Learning Satisfaction Scale (eLSS) (17 items) [31]. Responses to the eLSS are measured using a Likert scale, ranging from 1 (not at all) to 5 (strongly agree). Cronbach’s alpha for internal consistency reliability was 0.93.

### Other questions

The other questions will elicit self-reported information on general characteristics, including gestational age, number of children, gravidity, educational status, and employment status. Participants will be asked about their medical history of surgery and diseases, as well as any currently present diseases.

### Sample size

The sample size was calculated using G*Power 3.1.0 [32] with the following specifications: a one-tailed test; the independent-sample t-test; power, 0.70; effect size (f), 0.53; significance level of the one-sided test, 0.05; and a 1:1 allocation ratio [17]. The effect size (Cohen’s d) for the difference between the intervention and control group in primary outcomes was calculated based on a previous study [17]. The score for individual environmental behavior was 58.59 ± 12.25 in the intervention group and 51.93 ± 12.64 in the control group, resulting in a Cohen’s d of 0.53 [Cohen’s d = (51.93–58.59) ⁄12.446528 = 0.53]. The resulting sample size was determined to be 35 in each group. Even though the drop-out rate in the previous study was 5.8% (with a final total of 96 subjects out of 102 enrollments) [17], this study will adopt a more conservative rate of 10%. In conclusion, 40 subjects will be recruited for each group.

### Ethical considerations

This study was reviewed by the Institutional Review Board of Kongju National University (KNU-IRB-2022-010) and adhered to the Declaration of Helsinki. Women who want to participate in the study will complete and sign a written informed consent form.

### Statistical analysis

This study will use SPSS for Windows version 24.0 (IBM Corp., Armonk, NY, USA). The t-test and chi-square test will be used for homogeneity testing between the two groups. To test normality, linearity, and residual independence, the Shapiro-Wilk test will be used for the primary and secondary outcome variables. If the homogeneity test shows statistically non-significant differences between the two groups, the independent t-test will be used to compare the outcome variables between the groups. If the homogeneity test yields a statistically significant difference for even one independent variable, Analysis of covariance (ANCOVA) as a general linear model will be used to adjust the confounding variables. ANCOVA will test the following hypothesis: The intervention group would have higher scores for environmental health perceptions, behavior, psychological status, and quality of life after the DECO-MOM intervention than the control group.

## Discussion

This study will examine the effectiveness of a mobile application named DECO-MOM for pregnant women to enhance environmental health perceptions and behaviors as the primary outcome, and decrease depression and anxiety, increase quality of life, and e-learning satisfaction. The randomized controlled trial will enroll 80 subjects at two health care centers. This intervention will provide a valuable example of internet-based birth education for a vulnerable population to overcome the limitations of space and time [16]. As a mobile application, this program can be expanded to remote regions.

This intervention was carefully designed according to a theoretical basis. The primary outcomes of this study were derived from a theoretical framework according to the rPMT by Rogers [33]. Rogers argued that the concepts of reward and cost comprise an additional process for determining overall health behaviors, while severity, susceptibility, response efficacy, and self-efficacy are the main relevant concepts [33]. The inner cognitive mechanisms for determining environmental health behaviors, however, can be further examined in terms of the concept of cost. While severity and susceptibility may seem similar, severity refers to an individual’s perception of the harmfulness of health problems, whereas susceptibility refers to an individual’s perception of the likelihood that health problems will affect them [34]. Salazar [34] defined costs as the physical, social, and mental consequences of undertaking health behaviors. Therefore, it is necessary to emphasize environmental health issues from a macroscopic perspective and to present practical aspects of daily life and one’s own health to meet individuals’ needs from a microscopic perspective. The rPMT has been used in studies examining health information, accident prevention, political issues, and environmental issues [35], and it can be applied to modern environmental health issues and used in policies and studies that promote changes in environmental health behaviors for pregnant women.

A strength of this study will be that it analyzes the effectiveness of the intervention on secondary outcomes that reflect psychological aspects. An antecedent systematic review and meta-analysis of prenatal education using internet-based interventions was able to draw inferences regarding depression, anxiety, quality of life, and satisfaction [36]. Environmental pollution and environmental health factors, such as fine particulate matter, air pollution, heavy metals, and chemicals have been linked to depression, anxiety, and stress in the general population [37] and in pregnant women [38]. A web-based intervention was found to be more effective on depression and psychological well-being than a home-based intervention [39]. In previous studies, an internet-based intervention alleviated depression, but more validation was needed in the other psychological aspects, including anxiety [36]. Satisfaction was also affected by internet-based prenatal education [40]; hence, the secondary outcome variables were set in an evidence-based manner.

Another strength of this study will be that it minimizes confounding factors by conducting analysis of covariance using ANCOVA. This study will have a risk of bias from controlling the confounding variables of the educational effect. In order to offset this bias, appropriate statistical methods will be needed in the analysis process. Accounting for selection bias through homogeneity testing and covariate analysis will be required for participants’ characteristics observed before the start of education [41]. In the quality appraisal, intervention type, recruitment location, and the frequency, intensity, and timing of interventions should be clear and explicit at the protocol stage prior to the initiation of education [41]. Therefore, the educator should check that the intervention is implemented as intended, and manage the intervention so that the dropout rate between the intervention groups is not imbalanced or the dropout rate is excessively high.

This study will have several limitations. It will be a double-blinded randomized study; thus, the researchers will not be able contact pregnant women in person to provide social support given the need to guarantee anonymity. It is necessary to consider the risk of bias and report multiple effect estimates of the results [41]. The study population will be limited to participants capable of speaking and writing Korean in the light of cultural considerations, and to smart gadget owners; although smart gadget uptake is broad and nearly universal in Korea, economic exclusion will remain possible. Despite these limitations, this study will contribute to enhancing the environmental health conception and behaviors using a mobile application intervention, and it will serve as a valuable reference for future research.

### Trial status

The intervention is currently under development, and recruitment has not yet taken place.

## Data Availability

The data that support the findings of this study will be available from the corresponding author upon reasonable request.

## Abbreviations

ANCOVA: ANalysis of COVAriance
DECO-MOM: Deep ECOlogy-MOM
GAD-7: General Anxiety Disorder-7
e-LSS: e-Learning Satisfaction Scale
EPDS: Edinburgh Postpartum Depression Scale
EQ-5D: European Quality of life 5-Dimension
QR: Quick Response
rPMT: revised Protection Motivation Theory
TREND: Transparent Reporting of Evaluations with Nonrandomized Designs
WEHB: Women’s Environmental Health Behavior
WEHP: Women’s Environmental Health Perception
